# The association and prediction value of acylcarnitine on diabetic nephropathy in Chinese patients with type 2 diabetes mellitus

**DOI:** 10.1186/s13098-023-01058-1

**Published:** 2023-06-17

**Authors:** Xuerui Li, Yuyang Miao, Zhongze Fang, Qiang Zhang

**Affiliations:** 1 Department of Geriatrics, Tianjin Medical University General Hospital, Tianjin Geriatrics Institute, Anshan Road 154, Heping district, Tianjin, 300052 China; 2 Department of Toxicology and Sanitary Chemistry, School of Public Health, Tianjin Medical University, Qixiangtai Road 22, Heping district, Tianjin, 300070 China; 3 Tianjin Key Laboratory of Environment, Nutrition and Public Health, Tianjin, China

**Keywords:** Acylcarnitine, Diabetic nephropathy, Type 2 diabetes mellitus, Metabolism

## Abstract

**Background:**

Acylcarnitines play a role in type 2 diabetes mellitus (T2DM), but the relationship between acylcarnitine and diabetic nephropathy was unclear. We aimed to explore the association of acylcarnitine metabolites with diabetic nephropathy and estimate the predictive value of acylcarnitine for diabetic nephropathy.

**Methods:**

A total of 1032 (mean age: 57.24 ± 13.82) T2DM participants were derived from Liaoning Medical University First Affiliated Hospital. Mass Spectrometry was utilized to measure levels of 25 acylcarnitine metabolites in fasting plasma. Diabetic nephropathy was ascertained based on the medical records. Factor analysis was used to reduce the dimensions and extract factors of the 25 acylcarnitine metabolites. Logistic regression was used to estimate the relationship between factors extracted from the 25 acylcarnitine metabolites and diabetic nephropathy. Receiver operating characteristic curves were used to test the predictive values of acylcarnitine factors for diabetic nephropathy.

**Results:**

Among all T2DM participants, 138 (13.37%) patients had diabetic nephropathy. Six factors were extracted from 25 acylcarnitines, which account for 69.42% of the total variance. In multi-adjusted logistic regression models, the odds ratio (OR, 95% confidence interval [CI]) of diabetic nephropathy on factor 1 (including butyrylcarnitine/glutaryl-carnitine/hexanoylcarnitine/octanoylcarnitine/decanoylcarnitine/lauroylcarnitine/tetradecenoylcarnitine), factor 2 (including propionylcarnitine/palmitoylcarnitine/hydroxypalmitoleyl-carnitine/octadecanoylcarnitine/arachidiccarnitine), and factor 3 (including tetradecanoyldiacylcarnitine/behenic carnitine/tetracosanoic carnitine/hexacosanoic carnitine) were 1.33 (95%CI 1.12–1.58), 0.76 (95%CI 0.62–0.93), and 1.24 (95%CI 1.05–1.47), respectively. The area under the curve for diabetic nephropathy prediction was significantly increased after the complement of factors 1, 2, and 3 in traditional factors model (*P* < 0.01).

**Conclusions:**

Some plasma acylcarnitine metabolites extracted in factors 1 and 3 were higher in diabetic nephropathy, while factor 2 was lower in diabetic nephropathy among T2DM patients. The addition of acylcarnitine to traditional factors model improved the predictive value for diabetic nephropathy.

**Supplementary Information:**

The online version contains supplementary material available at 10.1186/s13098-023-01058-1.

## Background

In recent years, with the prolongation of the average life span of the population, the change in living habits, eating habits and structure, the prevalence of diabetes is on the rise [1, 2]. Besides, due to the improvement in treatment methods and the increase in survival time, the incidence of diabetes complications is also ascending [2]. Diabetic nephropathy is a common complication of diabetes mellitus and one of the serious manifestations of diabetic microvascular disease [3], which is a major cause of morbidity and mortality in patients with type 2 diabetes mellitus (T2DM) [4], leading to an important public health concern. Accordingly, it is necessary to identify early biomarkers to predict diabetic nephropathy and further implement early intervention strategies among T2DM patients.

Given that the pathogenesis of diabetic nephropathy is closely related to perturbed energy metabolism, studying the characteristic metabolic alterations in diabetic nephropathy may provide insights into better understanding of pathogenesis to identify new potential biomarkers and drug targets [5]. Acylcarnitine metabolites are a group of esters formed by the combination of free carnitine with acyl-coenzyme A (acyl-CoA) produced from fatty acids [6], besides, they are also important markers of peroxisome and mitochondrial oxidative disorders and can serve as biomarkers for metabolic syndrome. Some studies have indicated that acylcarnitines are related to T2DM, suggesting that accumulation of acylcarnitine could lead to imbalance in insulin synthesis and secretion, which further leads to β cell dysfunction [7, 8]. However, the relationship between acylcarnitine and diabetic nephropathy was unclear. Therefore, studies on the metabolic spectrum of acylcarnitine are important for understanding the development of diabetic nephropathy and its mechanisms.

Furthermore, a single biomarker, such as glycated hemoglobin (HbA1c) or glomerular filtration rate, alone or in combination, may not be sufficient to identify the subtle pathogenic pathways underlying complex diseases such as diabetic nephropathy. Some scholars used targeted metabolomics to study a group of acylcarnitine, evaluated their relationship with the incidence of diabetes, and incorporated them into the established T2DM risk model [9]. However, up to now, regarding whether and to what extent acylcarnitine predicts diabetic nephropathy is still unknown.

In the current study, we conducted a cross-sectional research to explore the association of acylcarnitine metabolites with diabetic nephropathy and estimate the predictive value of acylcarnitine for diabetic nephropathy in Chinese patients with T2DM.

## Methods

### Study population

The study population was derived from electronic medical records of 2,554 inpatients with available metabolite data from the main electronic database of Liaoning Medical University First Affiliated Hospital (LMUFAH), Jinzhou, China who were admitted to the hospital from May 2015 to August 2016, which was described in detail elsewhere [10]. In the current study, we focused on 1032 of these inpatients with T2DM.

#### Ethics approval

of the study was obtained from the Ethics Committee for Clinical Research of LMUFAH and informed consent was waivered by the Ethics Committee for Clinical Research of LMUFAH due to the retrospective nature of the cross-sectional study.

### Data collection

Data on demographic (age, sex), anthropometric characteristics (height, weight, and blood pressure), lifestyle (smoking status and alcohol consumption), clinical and laboratory measurements, duration of diabetes, and antidiabetic drug uses were obtained by electronic medical records. Body mass index (BMI) was calculated as weight in kilograms divided by height in meters squared (kg/m^2^). Blood pressure was measured in the sitting position after participants rested for at least 10-minute using a standard mercury sphygmomanometer. The clinical parameters included HbA1c, triglyceride, high density lipoprotein cholesterol (HDL-C), and low density lipoprotein cholesterol (LDL-C). According to HbA1c and lipid treatment goals recommended by the American Diabetes Association [11], HbA1c was categorized as < 7% and ≥ 7%, triglyceride was divided as < 1.7 mmol/L and ≥ 1.7 mmol/L, LDL-C was categorized as < 2.6 mmol/L and ≥ 2.6 mmol/L, HDL-C was categorized as < 1 mmol/L in male or < 1.3 mmol/L in female and ≥ 1 mmol/L in male or HDL-C ≥ 1.3 mmol/L in female. Antidiabetic drugs contained insulin and oral antidiabetic drugs (metformin, acarbose, sulfonylureas, thiazolidinediones, and glinides).

### Acylcarnitine quantification

The metabolomics assay method had been described in previous studies [12]. Briefly, dry blood spot samples collected by finger puncture after 8 h fasting were used for the metabolomics assay. The metabolomics in dry blood spots was measured using mass spectrometry (MS) technology. The MS metabolomic analysis was conducted using an AB Sciex 4000 QTrap system (AB Sciex, Framingham, MA, USA). The Electrospray ionization source was ion source. The ion spray voltage was 4.5 kV. Positive mode was performed to scan analytes. The mobile phase which carried the component to be tested was 80% acetonitrile aqueous solution. Isotope-labeled internal standards of acylcarnitine from Cambridge Isotope Laboratories (Tewksbury, MA, USA) were used for absolute quantification.

Specific acylcarnitine metabolites were as follows: acetylcarnitine (C2), propionylcarnitine (C3), butyrylcarnitine (C4), hydroxylbutyrylcarnitine (C4-OH), succinylcarnitine (C4DC), isovalerylcarnitine (C5), 3-hydroxyisovalerylcarnitine (C5-OH), glutarylcarnitine (C5DC), tiglylcarnitine (C5:1), hexanoylcarnitine (C6), octanoylcarnitine (C8), decanoylcarnitine (C10), lauroylcarnitine (C12), myristoylcarnitine (C14), 3-hydroxyltetradecanoylcarnitine (C14-OH), tetradecanoyldiacylcarnitine (C14DC), tetradecenoylcarnitine (C14:1), palmitoylcarnitine (C16), 3-hydroxypalmitoylcarnitine (C16-OH), 3-hydroxypalmitoleylcarnitine (C16:1-OH), octadecanoylcarnitine (C18), arachidic carnitine (C20), behenic carnitine (C22), tetracosanoic carnitine (C24) and hexacosanoic carnitine (C26).

### Assessment of diabetic nephropathy

Diabetic nephropathy was ascertained based on the medical records and defined as persistent albuminuria, progressive reduction in glomerular filtration rate, and hypertension judged by clinicians.

### Statistical analysis

The characteristics of T2DM patients with different diabetic nephropathy statuses were compared using t-test/Wilcoxon Rank Sum test for continuous variables and Chi-square tests for categorical variables.

Factor analysis was used to reduce a large number of correlated acylcarnitines to a smaller number of uncorrelated factors to cope with multiple comparisons, and the suitability for factor analysis was evaluated by Kaiser-Meyer-Olkin (KMO) and Bartlett sphericity tests [13]. A KMO coefficient around 0.8 was considered to be meritorious while a value < 0.5 was deemed to be unacceptable. Principal component analysis was used to extract factors and to obtain the corresponding factor loading matrix. Varimax rotation rotated the initial factor load matrix to obtain a solution that is more concise and easier to interpret results than the initial factor extraction [14]. Individual acylcarnitines that had the maximum loading for a factor were used as relevant components of the factor. Scree plot is a line plot of the eigenvalues of factors in factor analysis [15]. The horizontal axis of the scree plot is the number of factors, and the vertical axis is the eigenvalue of factors. The number of acylcarnitine factors was determined by eigenvalue, communalities, and scree plot: eigenvalue > 1, communalities ≥ 50%, and the number of factors located on the steep slope of the scree plot.

Multivariable binary logistic regression was used to estimate odds ratios (OR) and their 95% confidence intervals (CI) of the extracted acylcarnitine factors for diabetic nephropathy in T2DM. Age, sex, BMI, duration of diabetes, smoking, drinking, HbA1C, systolic blood pressure (SBP), triglyceride, HDL-C, LDL-C, and antidiabetic drugs were considered confounding. Areas under the receiver operating characteristic curves (AUC) were used to test the predictive values of traditional risk factors and specific acylcarnitine factors for the prevalence of diabetic nephropathy.

In the sensitivity analysis, we repeated the logistic regression models after multiple imputation of missing values in covariates. The level of statistical significance was set at a *P* value less than 0.05. All statistical analyses were performed using Statistical Analysis System Release 9.4 (SAS Institute Inc., Cary, NC, USA) and Stata SE 15.0 for Windows (StataCorp, College Station, Texas).

## Results

### Characteristics of the study population

Among all T2DM participants (n = 1,032), the mean age of patients was 57.24 ± 13.82 years, 483 (46.80%) were females, and the median duration of diabetes was 5 (Interquartile range: 0–10) years. Of them, 138 (13.37%) patients had diabetic nephropathy. Compared to T2DM patients without diabetic nephropathy, those with diabetic nephropathy were older and had longer duration of T2DM, higher SBP, HDL-C, and LDL-C, and higher use of antidiabetic drugs. There were no significant differences between two groups with respect to sex, BMI, smoking, drinking, and HbA1C (Table 1). Moreover, we further analyzed the association of the above-mentioned characteristics with diabetic nephropathy and found that longer duration of T2DM (OR 1.06, 95% CI 1.03–1.09), higher BMI (OR 1.07, 95% CI 1.01–1.14), and higher SBP (OR 1.01, 95% CI 1.00-1.02, *P* = 0.027) were associated with higher diabetic nephropathy prevalence. However, there was no statistical significance between other characteristics and diabetic nephropathy in the current study (Additional file 1: Table S1).

**Table 1:** Characteristic of the study population (n = 1,032)

	No-Diabetic nephropathy (n = 844)	Diabetic nephropathy (n = 138)	*P*
Age	56.82 ± 14.09	59.10 ± 12.37	0.027
Sex			0.517
Male	453 (53.67)	96 (51.06)	
Female	391 (43.33)	92 (48.94)	
Duration of diabetes	4.00 (0.00–10.00)	9.00 (2.00–15.00)	< 0.001
BMI	25.20 ± 3.84	25.70 ± 3.89	0.109
Smoking	268 (31.75)	63 (33.51)	0.641
Drinking	234 (27.73)	56 (29.79)	0.569
SBP	139.04 ± 23.43	146.56 ± 25.50	< 0.001
HbA1c	9.63 ± 2.41	9.50 ± 2.26	0.546
< 7%	57 (6.75)	20 (10.64)	< 0.001
≥ 7%	428 (50.71)	126 (67.02)	
Lack	359 (42.54)	42 (22.34)	
Triglyceride	2.03 ± 1.51	2.13 ± 1.80	0.496
< 1.7	315 (37.32)	68 (36.17)	0.106
≥ 1.7	284 (33.65)	77 (40.96)	
Lack	245 (29.03)	43 (22.87)	
HDL-C	1.07 ± 0.34	1.15 ± 0.39	0.01
≥ 1 in male or ≥ 1.3 in female	192 (22.75)	58 (30.85)	0.038
< 1 in male or < 1.3 in female	404 (47.87)	87 (46.28)	
Lack	248 (29.38)	43 (22.87)	
LDL-C	2.85 ± 0.97	3.05 ± 1.17	0.042
< 2.6	254 (30.09)	56 (29.79)	0.133
≥ 2.6	342 (40.52)	89 (47.34)	
Lack	248 (29.38)	43 (22.87)	
Antidiabetic drugs use	686 (81.28)	181 (96.28)	< 0.001

Data are presented as mean ± standard deviations, median (interquartile range), or number (proportion %)

BMI, body mass index; SBP, systolic blood pressure; HbA1c, glycated hemoglobin; HDL-C, high density lipoprotein cholesterol; LDL-C, low density lipoprotein cholesterol

In terms of acylcarnitine, C5DC, C14-OH, C22, and C26 were higher while C3 and C16 were lower in patients with diabetic nephropathy than their counterparts without diabetic nephropathy. Other acylcarnitines were similar between two groups (Additional file 1: Table S2).

### Extracted factors of acylcarnitines

**Fig. 1:**
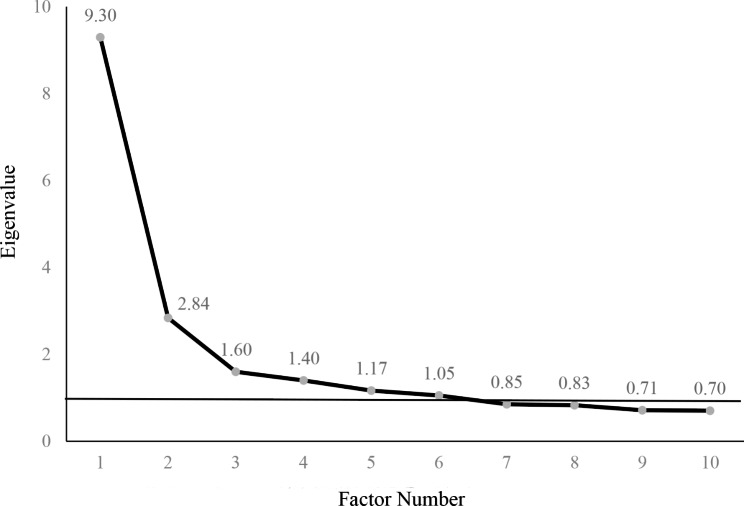
Eigenvalue of factors on scree plot The horizontal axis of the scree plot is the number of factors, and the vertical axis is the eigenvalue of factors. Six factors had eigenvalues of more than 1

Results of the factor analysis were acceptable as suggested by a high KMO coefficient of 0.882 and a highly significant *P*-value of Bartlett sphericity test of < 0.0001. Factors 1–6 had eigenvalues of more than 1 and were located on the steep slope of the scree plot (Fig. 1). Thus, six factors were extracted, and the loadings of acylcarnitines on them after varimax rotation were listed in Table 2. Factor 1 included C4, C5DC, C6, C8, C10, C12 and C14:1; Factor 2 contained C3, C16, C16:1-OH, C18 and C20; Factor 3 included C14DC, C22, C24 and C26; Factor 4 included C2 and C4-OH; Factor 5 included C4DC, C5, C5-OH and C5:1; Factor 6 included C14, C14-OH and C16-OH. The six factors explained 69.42% of the total variance.

**Table 2:** Factor and their loadings derived by 25 acylcarnitine metabolites

Variables	Factor1	Factor2	Factor3	Factor4	Factor5	Factor6
C2	0.50429	0.34748	0.14162	**0.68981**	0.10081	-0.02151
C3	0.08067	**0.53851**	0.21331	0.19845	0.45228	-0.09907
C4	**0.59019**	0.251	0.03502	0.53335	0.33466	-0.08973
C4-OH	0.07685	0.00693	0.07368	**0.82499**	0.00633	0.24523
C4DC	0.15497	0.01182	0.36127	-0.08586	**0.55835**	-0.07317
C5	0.25588	0.22914	0.10886	0.48798	**0.5693**	0.03655
C5-OH	0.02242	0.14218	0.1133	-0.01669	**0.70797**	0.33312
C5DC	**0.79163**	0.07983	0.16201	0.30658	0.05912	0.00873
C5:1	0.31919	0.17655	-0.00946	0.29204	**0.48242**	0.11599
C6	**0.8317**	0.16068	0.05042	0.30218	0.13862	0.02847
C8	**0.93584**	0.10313	0.05502	0.0886	0.08998	0.01758
C10	**0.89856**	0.09134	0.06551	-0.0812	0.09491	0.13032
C12	**0.73262**	0.20815	-0.01328	-0.09524	0.15774	0.44017
C14	0.43019	0.46226	-0.10485	0.00559	0.21252	**0.51255**
C14-OH	0.45857	0.09468	0.05866	0.18343	0.10609	**0.46761**
C14DC	0.40245	0.28539	**0.55178**	0.33555	0.00955	-0.047
C14:1	**0.80376**	0.19505	0.11113	0.11034	0.07487	0.14416
C16	0.2405	**0.83777**	0.06037	0.06701	0.13985	0.1017
C16-OH	0.08469	0.2005	0.18094	0.15398	0.07779	**0.69627**
C16:1-OH	0.14883	**0.68625**	0.31955	0.06626	0.08016	0.15477
C18	0.16894	**0.83086**	0.17011	0.08513	0.14699	0.15219
C20	0.07642	**0.48323**	0.32206	0.11856	-0.02242	0.16375
C22	-0.01123	0.2058	**0.69597**	0.32022	0.01519	0.21193
C24	0.09035	0.1236	**0.78191**	-0.03713	0.18574	0.16162
C26	0.03762	0.23012	**0.76184**	-0.04315	0.20975	-0.09637

C2, acetylcarnitine; C3, propionylcarnitine; C4, butyrylcarnitine; C4-OH, hydroxylbutyrylcarnitine; C4DC, succinylcarnitine; C5, isovalerylcarnitine; C5-OH, 3-hydroxyisovalerylcarnitine; C5DC, glutarylcarnitine; C5:1, tiglylcarnitine; C6, hexanoylcarnitine; C8, octanoylcarnitine; C10, decanoylcarnitine; C12, lauroylcarnitine; C14, myristoylcarnitine; C14-OH, 3-hydroxyl-tetradecanoylcarnitine; C14DC, tetradecanoyldiacylcarnitine; C14:1, tetradecenoylcarnitine; C16, palmitoylcarnitine; C16-OH, 3-hydroxypalmitoylcarnitine; C16:1-OH, 3-hydroxypalmitoleylcarnitine; C18, octadecanoylcarnitine; C20, arachidic carnitine; C22, behenic carnitine; C24, tetracosanoic carnitine; C26, hexacosanoic carnitine

The factors were extracted from 25 acylcarnitine according to eigenvalue > 1, scree plot, and cumulative variance by principal component analysis. Varimax rotation was used to maximize the variance difference of each factor, so as to better explain the factors. Each black-marked acylcarnitine in each column is classified as a factor in the corresponding column

### Relationship between extracted factors and diabetic nephropathy in T2DM

Factors 1 and 3 were significantly associated with higher diabetic nephropathy prevalence, while factor 2 was associated with lower diabetic nephropathy prevalence in univariate analysis. After adjustment for age and sex, the relationships of factors 1, 2 and 3 with the prevalence of diabetic nephropathy were not changed. In the multi-adjusted logistic model (i.e., adjustment for age, sex, BMI, duration of diabetes, smoking, drinking, SBP, HbA1C, triglyceride, LDL-C, HDL-C, and antidiabetic drug use), factor 1 (OR 1.33, 95% CI 1.12–1.58) and factor 3 (OR 1.24, 95% CI 1.05–1.47) were still associated with higher diabetic nephropathy prevalence, and factor 2 (OR 0.76, 95% CI 0.62–0.93) was associated with lower diabetic nephropathy prevalence. While, there was no statistically significant relationship between factor 4 (OR 0.99, 95% CI 0.84–1.16), factor 5 (OR 0.85, 95% CI 0.70–1.02), and factor 6 (OR, 1.12, 95% CI 0.94–1.33) and prevalence of diabetic nephropathy (Table 3).

**Table 3:** Odds ratios (ORs) and 95% confidence intervals (CIs) for the association between metabolomic factors with diabetic nephropathy

	Un-adjusted OR (95%CI)	Basic adjusted ^a^ OR (95%CI)	Muti-adjusted ^b^ OR (95%CI)
Factor1	1.28 (1.09–1.51)	1.25 (1.05–1.47)	1.32 (1.12–1.56)
Factor2	0.77 (0.64–0.92)	0.78 (0.65–0.94)	0.74 (0.60–0.91)
Factor3	1.23 (1.06–1.43)	1.29 (1.11–1.51)	1.25 (1.06–1.48)
Factor4	0.97 (0.81–1.16)	0.96 (0.78–1.17)	0.99 (0.84–1.16)
Factor5	0.85 (0.72-1.00)	0.89 (0.75–1.06)	0.85 (0.70–1.02)
Factor6	1.10 (0.94–1.28)	1.11 (0.95–1.30)	1.12 (0.94–1.33)

^a^ Adjusted for age and sex

^b^ Adjusted for age, sex, body mass index, duration of diabetes, smoking, drinking, glycated hemoglobin, systolic blood pressure, triglyceride, low density lipoprotein cholesterol, high density lipoprotein cholesterol, and antidiabetic drugs

### Predictive values for diabetic nephropathy in T2DM

**Fig. 2:**
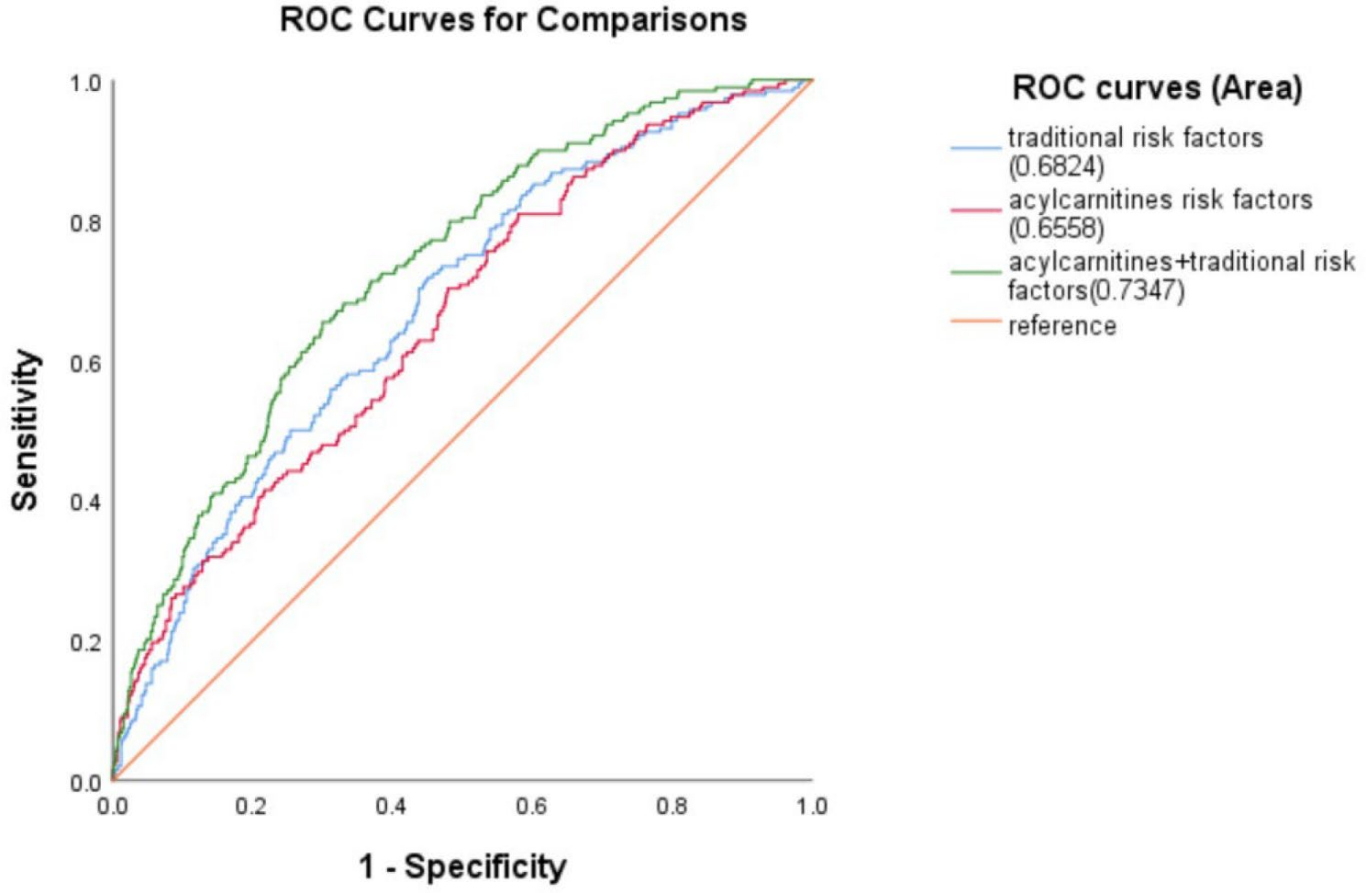
Receiver operating characteristic (ROC) curves of traditional risk factors, acylcarnitine factors, and traditional risk factors plus acylcarnitine factors for diabetic retinopathy in type 2 diabetes patients The blue curve stands for the traditional risk factor model; the red curve stands for acylcarnitine factors model; the green curve stands for the traditional risk factors plus the acylcarnitine factors model. The area under the operating characteristic curve was 0.6824 (95% CI 0.6409 to 0.7239) for the traditional risk factor model, 0.6558 (95% CI 0.6137 to 0.6980) for the acylcarnitine factors model, 0.7347 (95% CI 0.6969 to 0.7725) for the traditional risk factor plus acylcarnitine factors model (*P* < 0.05 for comparison of the traditional risk factor model with the traditional risk factor plus acylcarnitine factors model)

We established three models to test the predictive values of traditional risk factors, specific acylcarnitines, and their joint association for the prevalence of diabetic nephropathy. According to the results of logistic regression analysis, the specific acylcarnitines in this section only included those acylcarnitine indicators that were statistically associated with diabetic nephropathy. Model 1 was based on traditional risk factors for diabetic nephropathy, including age, sex, BMI, duration of diabetes, HbA1C, SBP, triglyceride, LDL-C, and HDL-C, as well as smoking and drinking status. Model 2 was based on specific acylcarnitines for diabetic nephropathy, including factor 1 (C4, C5DC, C6, C8, C10, C12 and C14:1), factor 2 (C3, C16, C16:1-OH, C18 and C20) and factor 3 (C14DC, C22, C24 and C26). Model 3 was based on all the variables of Model 1 and Model 2. The AUC of three models were 0.68 (95% CI: 0.64–0.72), 0.66 (95% CI: 0.61–0.70), and 0.73 (95% CI: 0.70–0.77), respectively (Fig. 2). *P* value was < 0.05 for comparison between Model 1 and Model 3.

### Supplementary analysis

The results were not materially altered compared to those from the initial analysis when we repeated the following analyses by multiple imputation of missing values in covariates (Additional file 1: Table S3).

## Discussion

In this population-based cross-sectional study, we found that some acylcarnitines, i.e., C4, C5DC, C6, C8, C10, C12 and C14:1 in factor 1 as well as C14DC, C22, C24 and C26 in factor 3, were higher in diabetic nephropathy, while factor 2 composed of C3, C16, C16:1-OH, C18 and C20 was lower in diabetic nephropathy among T2DM patients. Furthermore, the predicted value of diabetic nephropathy could be improved when acylcarnitines were added to the traditional factors model.

Given that the pathogenesis of diabetic nephropathy is closely related to perturbed energy metabolism, more and more scholars pay attention to the roles of metabonomics in diabetic nephropathy. Previous researches have shown acylcarnitines alteration in patients with T2DM and prediabetic condition compared to healthy people [16, 17], nevertheless, there are less data about carnitine deviations in diabetic nephropathy. Goek et al. completed a cross-sectional observation study including two independent samples, demonstrating that most acylcarnitines, especially C5DC, were associated inversely with glomerular filtration rate [18]. Furthermore, a cross-sectional study from Singapore showed that a number of short acylcarnitines including their dicarboxylic derivatives (C2-C6) were elevated in diabetic kidney disease, in particular, C4 was a positive, independent, and significant predictor of the albumin/creatinine ratio levels [19]. Another study by Esmati et al. reported that patients with severely increased albuminuria had a higher level of free carnitine, acylcarnitines including C2, C4, C5, C6, C8, C10, C14, C16 and C18 than patients with moderately increased albuminuria and normoalbuminuria did [20]. In the current study, we found that factors 1 and 3 were associated with a high prevalence of diabetic nephropathy (i.e., C4, C5DC, C6, C8, C10, C12, C14:1, C14DC, C22, C24 and C26 levels were higher in diabetic nephropathy), while factor 2 was associated with a low prevalence of diabetic nephropathy (i.e., C3, C16, C16:1-OH, C18 and C20 levels were lower in diabetic nephropathy). Possible explanations for the discrepancies between our study and others could be the differences in the use of acylcarnitines, stages of diabetic nephropathy, sample size and characteristics of study populations among these studies. Further large population-based prospective cohort studies are warranted to confirm our findings.

So far, the study regarding the predictive ability of acylcarnitine on diabetic nephropathy was limited. Ibarra et al. used a clinical metabonomics model to determine that changes in amino acid and acylcarnitine levels were associated with diabetic nephropathy, and the inclusion of metabonomics improved the predictive power of the clinical model to identify renal insufficiency and diabetic nephropathy related outcomes [21]. Abdelsattar et al. used MS and GC-MS to quantify the blood stains and urine samples of diabetic nephropathy patients and found that C12, C5:1 and C5 had stronger predictive abilities of albumin/creatinine values than HbA1c, suggesting that they could be used as potential biomarkers for the diagnosis of diabetic nephropathy at the early stage [22]. Our results also demonstrated that the inclusion of acylcarnitine metabolites, i.e., factors 1 (C4, C5DC, C6, C8, C10, C12 and C14:1), 2 (C3, C16, C16:1-OH, C18 and C20), and 3 (C14DC, C22, C24 and C26), improved the predictive capacity of diabetic nephropathy and the significance of studying metabolomics in the assessment of diabetic nephropathy in T2DM patients.

The potential mechanism of acylcarnitine on diabetic nephropathy development may include two pathways: impaired mitochondrial β-oxidation and complex lipid remodeling [23, 24]. The progression of diabetic nephropathy was related to impairments in the synthesis, desaturation, and oxidation of fatty acids. Acylcarnitine may accumulate in the plasma due to mitochondrial damage, and its concentration may indicate the rate of β-oxidation of fatty acids in diabetic nephropathy progression [21, 25]. Therefore, theoretically, all short-, medium- and long-chain acylcarnitine would be elevated in patients with diabetic nephropathy. While, although fatty acids oxidation may initiate adaptive compensation (fatty acids prolongation and desaturation) at early stage, as the degree of diabetic nephropathy progressively progresses, it is difficult to compensate for the renal metabolic and pathological changes by intensive fatty acids oxidation. Thus, the incomplete β-oxidation of long-chain fatty acids is increased, resulting in an increased plasma short- and medium-chain acylcarnitines and a decreased long-chain acylcarnitines [26]. In addition, lipid metabolic changes may be associated with diabetic nephropathy, such as activation of acetyl-CoA carboxylase (ACC), which was a key determinant of diabetic nephropathy progression. Upregulation of ACC inhibited CTPI, which in turn inhibited the cytoplasmic conversion of long-chain acyl-CoA to long-chain acylcarnitine, thereby reducing the substrate for the carnitine shuttle, affecting the β-oxidation of long-chain fatty acids, and increasing the abundance of cytoplasmic palmitic acid [26]. Furthermore, a large amount of acylcarnitine, especially C4, entered the tricarboxylic acid cycle, thereby inhibiting glucose oxidation, which may further exacerbate the intracellular demand for carnitine stores [19].

The strength of our study is the use of a relatively large number of acylcarnitines and the classification of acylcarnitines index with dimensionality reduction by factor analysis. However, there were several limitations noticed in our study. First, the cross-sectional design of the current study could not establish causality between acylcarnitines and diabetic nephropathy in T2DM, and large population-based prospective cohort studies are needed to confirm our findings. Furthermore, the participants in the current study were inpatients with diabetic nephropathy, who were more serious, thus caution is needed when generalizing our findings to other populations.

## Conclusions

Our study found that some plasma acylcarnitine metabolites extracted in factors 1 and 3, including C4, C5DC, C6, C8, C10, C12, C14:1, C14DC, C22, C24 and C26, were associated with higher prevalence of diabetic nephropathy, while some acylcarnitine extracted in factor 2, i.e., C3, C16, C16:1-OH, C18 and C20, were lower in diabetic nephropathy among T2DM patients. The addition of acylcarnitine to traditional factors model improved the predictive value for diabetic nephropathy. Our findings underscore the importance of acylcarnitines in diabetic nephropathy and, of course, future prospective cohort studies are needed to replicate our findings.

## Electronic supplementary material

Below is the link to the electronic supplementary material.

Additional file 1
Table S1 Odds ratios (ORs) and 95% confidence intervals (CIs) for the association between population characteristic factors with diabetic nephropathy.
Table S2. Acylcarnitine profile in T2DM patients (n = 1,032). Table S3. Odds ratios (ORs) and 95% confidence intervals (CIs) for the association between metabolomic factors with diabetic nephropathy by multiple imputation of missing values in covariates.

## Data Availability

The datasets generated during and/or analysed during the current study are available from the corresponding author on reasonable request.

## List of Abbreviations

acyl-CoA: acyl-coenzyme A
ACC: acetyl-CoA carboxylase
AUC: Area under curve
BMI: Body mass index
C2: Acetylcarnitine
C3: Propionylcarnitine
C4: Butyrylcarnitine
C4-OH: Hydroxylbutyrylcarnitine
C4DC: Succinylcarnitine
C5: Isovalerylcarnitine
C5-OH: 3-hydroxyisovalerylcarnitine
C5DC: Glutarylcarnitine
C5:1: Tiglylcarnitine
C6: Hexanoylcarnitine
C8: Octanoylcarnitine
C10: Decanoylcarnitine
C12: Lauroylcarnitine
C14: Myristoylcarnitine
C14-OH: 3-hydroxyltetradecanoylcarnitine
C14DC: Tetradecanoyldiacylcarnitine
C14:1: Tetradecenoylcarnitine
C16: Palmitoylcarnitine
C16-OH: 3-hydroxypalmitoylcarnitine
C16:1-OH: 3-hydroxypalmitoleylcarnitine
C18: Octadecanoylcarnitine
C20: Arachidic carnitine
C22: Behenic carnitine
C24: Tetracosanoic carnitine
C26: Hexacosanoic carnitine
CI: Confidence interval
HbA1c: Glycated hemoglobin
HDL-C: High density lipoprotein cholesterol
KMO: Kaiser-Meyer-Olkin
LDL-C: Low density lipoprotein cholesterol
LMUFAH: Liaoning Medical University First Affiliated Hospital
OR: Odds ratio
SBP: Systolic blood pressure
T2DM: Type 2 diabetes mellitus
