# Supplementary material for: The association and prediction value of acylcarnitine on diabetic nephropathy in Chinese patients with type 2 diabetes mellitus
Source: Diabetol Metab Syndr. 2023 Jun 17;15:130. doi: 10.1186/s13098-023-01058-1 (PMC10276375; doi:10.1186/s13098-023-01058-1)
Supplement: Supplementary file 1 — Additional file 1 Table S1 Odds ratios (ORs) and 95% confidence intervals (CIs) for the association between population characteristic factors with diabetic nephropathy. Table S2. Acylcarnitine profile in T2DM patients (n = 1,032). Table S3. Odds ratios (ORs) and 95% confidence intervals (CIs) for the association between metabolomic factors with diabetic nephropathy by multiple imputation of missing values in covariates. [file 13098_2023_1058_MOESM1_ESM.docx]

**Supplementary Material**

**Table S1**. Odds ratios (ORs) and 95% confidence intervals (CIs) for the association between population characteristic factors with diabetic nephropathy.

**Table S2**. Acylcarnitine profile in T2DM patients (n=1,032).

**Table S3**. Odds ratios (ORs) and 95% confidence intervals (CIs) for the association between metabolomic factors with diabetic nephropathy by multiple imputation of missing values in covariates.

**Table S1**. Odds ratios (ORs) and 95% confidence intervals (CIs) for the association between population characteristic factors with diabetic nephropathy.

|  | Multi-adjusted OR (95%CI) |
| --- | --- |
| Age | 1.01 (0.99-1.02) |
| Female | 1.37 (0.80-2.36) |
| Duration of diabetes | 1.06 (1.03-1.09) |
| Body mass index | 1.07 (1.01-1.14) |
| Smoking | 0.99 (0.56-1.74) |
| Drinking | 1.35 (0.74-2.46) |
| Systolic blood pressure | 1.01 (1.00-1.02) |
| Glycated hemoglobin | 1.02 (0.92-1.12) |
| Triglyceride | 1.02 (0.90-1.16) |
| High density lipoprotein cholesterol | 1.28 (0.69-2.37) |
| Low density lipoprotein cholesterol | 0.96 (0.77-1.20) |
| Antidiabetic drugs | 2.65 (0.90-7.85) |

Model was adjusted for age, sex, duration of diabetes, body mass index, smoking, drinking, systolic blood pressure, glycated hemoglobin, triglyceride, high density lipoprotein cholesterol, low density lipoprotein cholesterol, and antidiabetic drugs.

**Table S2**. Acylcarnitine profile in T2DM patients (n=1,032).

|  | No-Diabetic nephropathy  (n=844) | Diabetic nephropathy  (n=138) | *P* |
| --- | --- | --- | --- |
| C2 | 11.615 (8.848-15.680) | 11.405 (8.662-14.477) | 0.259 |
| C3 | 1.380 (0.968-1.970) | 1.257 (0.890-1.747) | 0.013 |
| C4 | 0.200 (0.151-0.280) | 0.209 (0.150-0.270) | 0.862 |
| C4-OH | 0.108 (0.079-0.159) | 0.100 (0.076-0.150) | 0.348 |
| C4DC | 0.649 (0.480-0.860) | 0.640 (0.500-0.786) | 0.516 |
| C5 | 0.149 (0.110-0.193) | 0.146 (0.110-0.204) | 0.886 |
| C5-OH | 0.270 (0.200-0.356) | 0.245 (0.181-0.340) | 0.077 |
| C5DC | 0.080 (0.050-0.118) | 0.092 (0.054-0.131) | 0.037 |
| C5:1 | 0.060 (0.048-0.080) | 0.060 (0.046-0.073) | 0.149 |
| C6 | 0.050 (0.035-0.068) | 0.050 (0.036-0.075) | 0.230 |
| C8 | 0.067 (0.044-0.100) | 0.071 (0.049-0.104) | 0.319 |
| C10 | 0.090 (0.060-0.150) | 0.090 (0.060-0.180) | 0.355 |
| C12 | 0.051 (0.040-0.070) | 0.055 (0.040-0.078) | 0.482 |
| C14 | 0.068 (0.050-0.090) | 0.061 (0.050-0.085) | 0.454 |
| C14-OH | 0.050 (0.040-0.070) | 0.055 (0.004-0.072) | 0.037 |
| C14DC | 0.040 (0.030-0.059) | 0.040 (0.030-0.059) | 0.803 |
| C14:1 | 0.095 (0.070-0.130) | 0.98 (0.070-0.129) | 0.638 |
| C16 | 0.930 (0.720-1.180) | 0.843 (0.701-1.090) | 0.023 |
| C16-OH | 0.026 (0.020-0.037) | 0.029 (0.020-0.040) | 0.364 |
| C16:1-OH | 0.055 (0.040-0.070) | 0.052 (0.040-0.070) | 0.327 |
| C18 | 0.449 (0.357-0.570) | 0.423 (0.338-0.564) | 0.247 |
| C20 | 0.047 (0.038-0.060) | 0.046 (0.038-0.060) | 0.925 |
| C22 | 0.075 (0.060-0.096) | 0.080 (0.060-0.102) | 0.031 |
| C24 | 0.056 (0.040-0.070) | 0.060 (0.040-0.075) | 0.193 |
| C26 | 0.035 (0.027-0.049) | 0.040 (0.030-0.052) | 0.029 |

Data are presented as median (interquartile range).

C2, acetylcarnitine; C3, propionylcarnitine; C4, butyrylcarnitine; C4-OH, hydroxylbutyrylcarnitine; C4DC, succinylcarnitine; C5, isovalerylcarnitine; C5-OH, 3-hydroxyisovalerylcarnitine; C5DC, glutarylcarnitine; C5:1, tiglylcarnitine; C6, hexanoylcarnitine; C8, octanoylcarnitine; C10, decanoylcarnitine; C12, lauroylcarnitine; C14, myristoylcarnitine; C14-OH, 3-hydroxyl-tetradecanoylcarnitine; C14DC, tetradecanoyldiacylcarnitine; C14:1, tetradecenoylcarnitine; C16, palmitoylcarnitine; C16-OH, 3-hydroxypalmitoylcarnitine; C16:1-OH, 3-hydroxypalmitoleylcarnitine; C18, octadecanoylcarnitine; C20, arachidic carnitine; C22, behenic carnitine; C24, tetracosanoic carnitine; C26, hexacosanoic carnitine.

**Table S3**. Odds ratios (ORs) and 95% confidence intervals (CIs) for the association between metabolomic factors with diabetic nephropathy by multiple imputation of missing values in covariates.

|  | Muti-adjusted OR (95%CI) |
| --- | --- |
| Factor1 | 1.23 (1.07-1.42) |
| Factor2 | 0.76 (0.64-0.92) |
| Factor3 | 1.28 (1.09-1.50) |
| Factor4 | 0.96 (0.83-1.12) |
| Factor5 | 0.87 (0.73-1.04) |
| Factor6 | 1.12 (0.95-1.31) |

Adjusted for age, sex, body mass index, duration of diabetes, smoking, drinking, glycated hemoglobin, systolic blood pressure, triglyceride, low density lipoprotein cholesterol, high density lipoprotein cholesterol, and antidiabetic drugs.
